# Esculetin releases maturation arrest and induces terminal differentiation in leukemic blast cells by altering the Wnt signaling axes

**DOI:** 10.1186/s12885-023-10818-1

**Published:** 2023-05-01

**Authors:** Ankit Mathur, Aman Gangwar, Daman Saluja

**Affiliations:** 1 Delhi School of Public Health, Institution of Eminence, University of Delhi, Delhi, 110007 India; 2 Dr. B.R. Ambedkar Center for Biomedical Research, University of Delhi, Delhi, 110007 India

**Keywords:** Acute Myeloid Leukemia (AML), Esculetin, Blast cell differentiation, Differentiation therapy, Kasumi-1, Wnt signaling

## Abstract

**Background:**

The “Differentiation therapy” has been emerging as a promising and more effective strategy against acute leukemia relapses.

**Objective:**

In extension to the revolutionising therapeutic outcomes of All Trans Retinoic Acid (ATRA) to induce terminal differentiation of Acute Promyelocytic Leukemic (APL) blast cells, we decipher the potential effect of a natural compound “Esculetin” to serve as a differentiating agent in Acute Myeloid Leukemia (AML). Underlaying role of Wnt signaling pathways in esculetin mediated blast cell differentiation was also evaluated.

**Methods:**

Human acute myeloid leukemic cells (Kasumi-1) with t(8;21/AML-ETO) translocation were used as a model system. Growth inhibitory and cytotoxic activity of esculetin were analysed using growth kinetics and MTT assay. Morphological alterations, cell scatter characteristics, NBT reduction assay and cell surface marker expression patterns were analysed to detect terminally differentiated phenotypes. We employed RT^2^profiler PCR array system for the analysis of transcriptome profile of Wnt signaling components. Calcium inhibitors (TMB8 and Amlodipine) and Transforming growth factor beta (TGF-β) were used to modulate the Wnt signaling axes.

**Results:**

We illustrate cytotoxic as well as blast cell differentiation potential of esculetin on Kasumi-1 cells. Morphological alterations akin to neutrophilic differentiation as well as the corresponding acquisition of myeloid lineage markers indicate terminal differentiation potential of esculetin in leukemic blast cells. Exposure to esculetin also resulted in downregulation of canonical Wnt axis while upto ~ 21 fold upregulation of non-canonical axis associated genes.

**Conclusions:**

Our study highlights the importance of selective use of calcium pools as well as “axis shift” of the canonical to non-canonical Wnt signaling upon esculetin treatment which might abrogate the inherent proliferation to release maturation arrest and induce the differentiation in leukemic blast cells. The current findings provide further therapeutic interventions to consider esculetin as a potent differentiating agent to counteract AML relapses.

**Supplementary Information:**

The online version contains supplementary material available at 10.1186/s12885-023-10818-1.

## Introduction

AML is a heterogeneous malignancy attributed to impaired clonal evolution of hematopoietic cells. The genetic and hierarchical complexity within the AML subpopulations results in a pronounced phenotypic variability in which myeloid precursor cells acquire proliferative advantages while abrogating the terminal differentiation potential [1, 2]. The current cytotoxic therapies target to eradicate the outcompeting leukemic immature blast population. Despite therapeutic improvements, the persistence of multi-drug resistant clones leading to minimal residual disease (MRD) is a major hurdle in achieving the complete remission [3]. Therefore, alternative approaches are required to be considered for reducing the recurrence and expansion of the residual disease. Recently, a therapeutic strategy called “Differentiation Therapy” directed towards removing the maturation block and allowing blast cell differentiation appears to be a more specific and effective therapy than traditional approaches with cytotoxic drugs [4]. A remarkable example of the differentiating agent is All-Trans Retinoic Acid (ATRA), which has not only revolutionized the therapeutic outcomes in patients with Acute Promyelocytic Leukemia (APL), but also have pave the way to alter the landscape in AML treatment. However, the early promises of differentiation therapy could not be translated for AML and the conventional chemotherapeutic regimens remained the standard of care.

Natural Compounds have emerged as a rich and attractive source of therapeutics to serve as potent antitumor agents with high selectivity for tumour cells. We have previously demonstrated that “Esculetin”, a dihydroxycoumarin derivative induces pro-apoptotic and anti-proliferative activity in leukemic (Kasumi-1) as well as human pancreatic cancer cell line (PANC-1). We also demonstrated that the anti-leukemic effects of esculetin on Kasumi-1 cells was attributed by its ability to reduce the half-life of AML1-ETO and c-Kit m-RNAs [5, 6]. These studies have prompted us to hypothesise that esculetin might have a potential to selectively differentiate the leukemic blast cells. Nearly twenty percent of AML patients carry a translocation between chromosomes 21 and chromosome 8 resulting in the formation of a chimeric oncoprotein AML1-ETO; promoting self-renewal and blocking myeloid differentiation and poor prognosis. It is anticipated that identification of novel therapeutic targets in t(8;21) positive AML will lead to treatment options that improve patient survival [7].

Among plethora of signaling mechanisms, the Wnt signaling have emerged as a key player in maintaining hematopoietic stem cell renewal, cell proliferation as well as differentiation [8]. Moreover, aberrant Wnt signaling has been found to be associated with early induction of leukemogenesis and maintenance of leukemic blast/stem cell pool [9, 10]. More recently, the regulatory components and the cross-talks of both the canonical-and non-canonical Wnt signaling is taking a central role in the leukemogenesis [11, 12]. Therefore, in the current study using Kasumi-1 cells as a model system, we aim to decipher the potential of esculetin to induce differentiation in leukemic blast cells and the possible involvement of Wnt signaling in esculetin mediated blast cell differentiation.

## Material and methods

### Cell culture and maintenance

Human Myeloid Leukemic Kasumi-1 cell line was obtained from National Centre for Cell Science (Pune, India) and were maintained in suspension at 37^O^C in RPMI 1640 supplemented with 10% fetal bovine serum, 2 mM Glutamine and antibiotics (Penicillin-50,000 Units/L, Streptomycin- 50 µg/ml) in a humidified incubator.

### Cell proliferation assay

Exponentially growing Kasumi-1 cells were equally (1 × 10^5^ cells) seeded onto 6-well plate in complete growth medium in the presence and/or absence of esculetin. The cells were counted using a Neubouer hemocytometer up to 96 h at 24 h time intervals.

### MTT Assay for cytotoxicity

To assess the cytotoxic response of esculetin on Kasumi-1 cells, we performed 3-(4,5-dimethylthiazol-2-yl)-5-(3-carboxymethoxyphenyl)-2-(4-sulfophenyl)-2H-tetrazolium (MTT; Himedia Laboratories) assay. 2 × 10^5^ cells/well were plated into a 6-well plate. At log phase in culture, cells were treated with defined concentrations of esculetin ranging from 25 µM to 200 µM and incubated at varied time intervals. MTT was then added and cells were incubated for three hours and the formazan crystals were dissolved in DMSO. The absorbance was measured at 570 nm using an enzyme-linked immunosorbent assay (ELISA) reader (Tecan spark control) instrument. The IC50 was calculated using AAT bioquest software.

### Cell cycle analysis

The effect of esculetin on cell cycle distribution at varied time intervals was evaluated using Propidium Iodide (PI) method. Cells were resuspended in PBS and fixed in 70% chilled ethanol overnight. Following PBS washing, RNAse A (100 µg/ml) was added and cells were incubated at 37 C for 30 mints. Propidium Iodide (10 µg/ml) was incorporated directly 15 min before acquisition by FACS Calibur (BD Biosciences).

### Apoptosis detection by flow cytometry

Apoptotic cell death was analysed by Annexin V and Propidium Iodide double staining following 24/48 h of esculetin treatment according to the manufacturer’s instructions (Invitrogen) using FACSCalibur and Flowing software.

### Nitroblue Tetrazolium Test (NBT Assay) for cell differentiation

NBT assay is used to determine the ability of cells to produce reactive oxygen species and provide insight into their oxidative metabolism. Since the generation of ROS has been associated with a myroid of stress/pathologic conditions, Phorbol 12-Myristate 13-Acetate (PMA /200 ng/ml) was used as a stress inducer. The PMA treated cells were subjected to esculetin treatment followed by incubating the cells with 1% NBT for three hours. Microscopically, 500 cells with intact morphology were analysed for the presence of intracellular formazan crystal formation in control verses esculetin treated cells. Equal number of live cells were taken and precision of the above microscopic technique was confirmed by colorimetric assay (650 nm) by disolving the formazan crystals in Dimethyl Sulfoxide (DMSO).

### Cell surface marker analysis

Following 96 h esculetin treatment, approximately 1–2 × 10^6^ cells were resuspended in PBS containg 0.5% Bovine Serum Albumin (BSA) for 1 h to inhibit non-specific antibody binding. Cells were stained with FITC anti-CD34/ PE-anti-CD38 and/or anti-CD11b (Abcam) antibodies. Respective fluorescently labblled secondary antibodies for non-conjugated antibodies (Abcam)were used to detect the expression of neutrophil/monocytic specific markers. Stained cells were then analysed by FACScalibour flowcytometer.

### Real-Time PCR for gene expression analysis

Total RNA was isolated from Kasumi-1 cells treated with/or without esculetin using RNeasy kit (Qiagen). Following cDNA conversion, RT-PCR of the selected genes was performed using SYBR Green Master (ThermoFisher Scientific) on QuantStudio 7 Flex Sytem (Applied Biosystems). The fold change of the gene expression was analysed using 2^−ΔΔ^CT method. β-actin was used as an endogenous control. Sequence of specific primers is shown in Supplementary Table 1.

### Western blotting for protein expression analysis

Western blotting was used to determine the alterations in protein expressions. Anti- β-catenin and anti- β-actin antibodies (Santa Cruz Biotechnology) were used for respective protein detection. BSA was used as a blocking agent. Horseradish peroxidase-conjugated secondary antibodies (Santa Cruz) along with a chemiluminescence detection system (Bio-Rad) were used for the detection of protein bands. Quantification was carried out using ImageQuant system (GE Healthcare).

### Morphological analysis by Giemsa staining

The cytospins of Giemsa (Sigma-Aldrich) stained, esculetin treated /untreated cells were prepared and images were captured using Nikon eclipse TI2 microscope (Nikon). To quantify the percent nuclear alterations, nuclear morphology of 500 randomly selected cells were analysed.

### Intracellular calcium assessment

Cells were washed with PBS followed by incubation of 1 µM Fluo-4/AM (Invitrogen) for 30 min at 37^0^C in dark. Cells were then washed with PBS before analysis using FACSCalibur (BD).

### Intracellular localization of β-catenin

Esculetin treated/untreated cells were fixed with 75% methanol for 10 min. After fixation, cells were washed with PBS and permeabilized for 10 min with 0.05% Triton- × 100. After blocking with 1% FBS for 1 h, cells were probed with β-catenin antibody. Cells were washed with PBS containing 0.1% Tween-20 and probed with FITC labeled IgG antibody for 1 h. Nucleus was stained with 4’,6-diamidino-2-phenylindole (DAPI). Images were captured using fluorescence microscope (Axio Imager.M2; Carl Zeiss) and processed using AxioVision Rel.4.8 software (Carl Zeiss).

### Statistical analysis

The flowcytometry analysis was performed using Flowing software. Microsoft excel was used to calculate the differences in mean (± SE) values of different treatments and statistical significance was determined using unpaired two-tailed Student's t-test for independent samples.

## Results

### Esculetin induces growth inhibitory and cytotoxic effects in Kasumi-1 cells

**Fig. 1:**
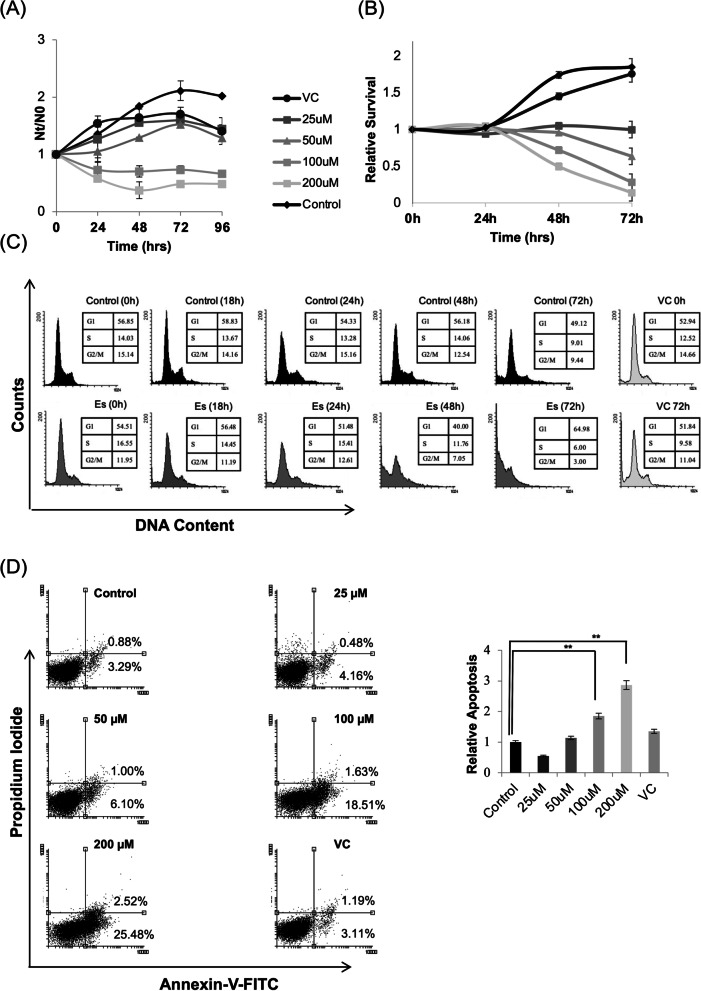
Growth inhibitory and cytotoxic effects esculetin. **A** Proliferation kinetics of Kasumi-1 cells treated/untreated with esculetin. The cells were treated with different concentrations of esculetin (25,50,100,200,500 µM) and counted at indicated time intervals. DMSO treated cells served as controls. Representative data of three independent experiments were plotted. **B** Cell viability was determined using MTT assay. Cells were treated with DMSO alone (Vehicle Control/VC) or with increasing concentrations of esculetin upto 72 h and analysed for survival using MTT assay. Relative cell survival to DMSO treated cells is shown. **C** Cell cycle distribution analysis of Kasumi-1 cells in presence or absence of esculetin (100 μM) and VC at mentioned time intervals using flow cytometer. The relative DNA content of PI-stained cells shows cells at different phases of the cell cycle. **D** Representative dot plot analysis of esculetin mediated apoptosis based on Annexin V and propedium iodide (PI) staining. Annexin V positive cells were considered to undergo early apoptosis, and Annexin V + PI positive cells as late apoptotic cells. Percentage cell population in each quardant and relative apoptosis in bar graph (right panel) are combined from three independent experiments. (*p ≤ 0.05; **p ≤ 0.02)

In order to determine the effect of esculetin on the growth of Kasumi-1 cells, we treated the cells with varying concentrations (0, 25, 50, 100 and 200 µM) of esculetin. We measured a concentration and time dependent suppression of growth of Kasumi-1 cells upon esculetin treatment (Fig. 1A). Esculetin could not exert a noticeable growth inhibitory activity up to 50 µM concentration at studied time points. However, the growth suppression was more pronounced at esculetin concentration of 100 µM and above. The cell viability of DMSO (vehicle control/VC) and untreated cells were comparable and the difference between them was found to be insignificant.

The cytotoxicity was measured using MTT assay. Esculetin was able to induce cytotoxicity in Kasumi-1 cells in a dose and time dependent manner (Fig. 1B). The Inhibitory Concentration (IC_50_) was calculated at 100 µM. It is to be noted that the difference between cell viability of DMSO treated cells and untreated cells was insignificant.

To further assess whether the growth inhibitory and cytotoxic effect of esculetin involves altered cell cycle distribution, PI-stained cells were subjected to cell cycle analysis at varied time intervals (18 h, 24 h, 48, 72 h) following 100 μM esculetin treatment. As shown in Fig. 1(C), Kasumi-1 cells exhibited a higher G1 population as compared to control cells immediately following 24 h of esculetin treatment. Moreover, a concomitant increase in the proportion of G1/S while a significant reduction in G2/M populations was detected after 48 h which persisted at 72 h following esculetin treatment. These observations indicate that esculetin induces a gradual G1/S block which was prominent at 48 h and onwards following esculetin treatment.

With a higher proportion of hypodiploid DNA content in the cell cycle analysis, flowcytometric annexinV-FITC/PI assay further revealed a reduction in percentage of viable cells in a dose and time dependent manner (Fig. 1D). Esculetin at 100 µM triggered cells to undergo early apoptosis (~ four fold) 24 h post treatment (Supplementary Fig. 1) which gradually increased up-to 48 h with nearly all doses in dose dependent manner (Fig. 1D). Cells undergoing necrosis (PI positive cells) could not be detected at the observed time point. However, a marked percentage of necrotic cells detected at 25 µM of esculetin treatment are indicative of preferential induction of necrosis rather than apoptosis at this concentration.

### Exposure to esculetin promotes differentiation in leukemic precursor/blast cells

**Fig. 2:**
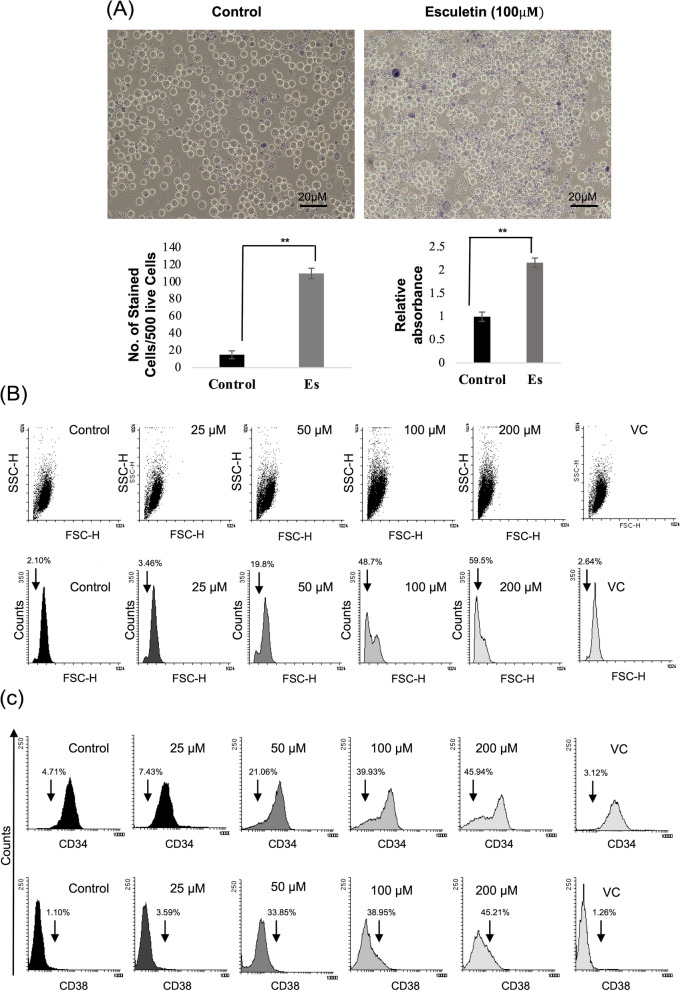
Blast cell differentiation potential of esculetin in Kasumi-1 cells. **A** Microscopic analysis of NBT stained cells. Exponentially growing Kasumi-1 cells treated with/without esculetin (100 µM) for 96 h. PMA was used for ROS stimulation and MTT was added. Manual counting (Lower left panel) and colorimetric analysis of dissolved crystals in DMSO (lower right panel) of treated/untreated cells was carried out to determine relative staining using NBT reduction assay (**p ≤ 0.02). **B** Flow cytometric analysis of unfixed/unstained Kasumi-1 cells treated with varied esculetin concentrations to measure cellular granularity (FSC versus SSC) (Upper panel) and cell size (FSC verses cell count) (Lower Panel). High side scatter (SSC) indicative of cells with enhanced granularity. A dose dependent left shift of the forwarded scatter (FSC) upon esculetin treatment indicative of reduction in cell size. Scatter characteristics of 10,000 cells were analysed. Percentage of shifted cells was measured using flowing software. **C** Surface expression analysis of immaturity markers (CD34) (Upper Panel) and maturity marker (CD38) (Lower panel) using flow cytometry following 96 h of esculetin treatment. Percentage of shifted cells was measured using flowing software

The differentiation potential was assesed using NBT reduction assay to detect the ability of differentiated cells to produce reactive oxygen species; a property of terminally differentiated myeloid cells. PMA stimulated/esculetin-treated cells demonstrated a high cytoplasmic formazan deposits than untreated cells (Fig. 2A). The above manual cell counting assay was further validated using colorimetric assay by dissolving the formazan crystals in DMSO. A nearly twofold higher absorbance at 650 nm was determined in esculetin-treated cells as compared to the control cells.

During hematopoietic blast cell differentiation, sequential maturation of immature cells involves homeostatic control mechanisms that regulates the cell size and granularity. Esculetin-treated Kasumi-1 cells exhibited a relatively smaller cell size during microscopic evaluation. Therefore, scatter characteristics of unfixed, unstained Kasumi-1 cells were analyzed to detect the cell size (Forward scatter) versus granularity (side scatter) upon esculetin treatment using flow cytometry. A gradual reduction in cell size (left shift of FSC) while increased side scatter characteristics indicate generation of relatively smaller and highly granular cells upon esculetin teatment as compared to control cells (Fig. 2B). We found upto ~ 49% population at 100 µM esculetin concentration had reduced cell size as comaperd to control cells. DMSO alone at used concentration was not found to exert any effect on cell size.

The above differentiation features corresponded with the altered cell surface marker expression profile. A gradual reduction in CD34^+^ (Immaturity marker) in ~ 40% of population at 100 μM while enhanced CD38^+^ (maturity marker) in ~ 39% of population was observed following 96 h of esculetin treatment reflecting the differentiation potential of esculetin on Kasumi-1 cells (Fig. 2C).

### Morphological and marker expression alterations indicative of neutrophil differentiation in Kasumi-1 cells upon esculetin exposure

**Fig. 3:**
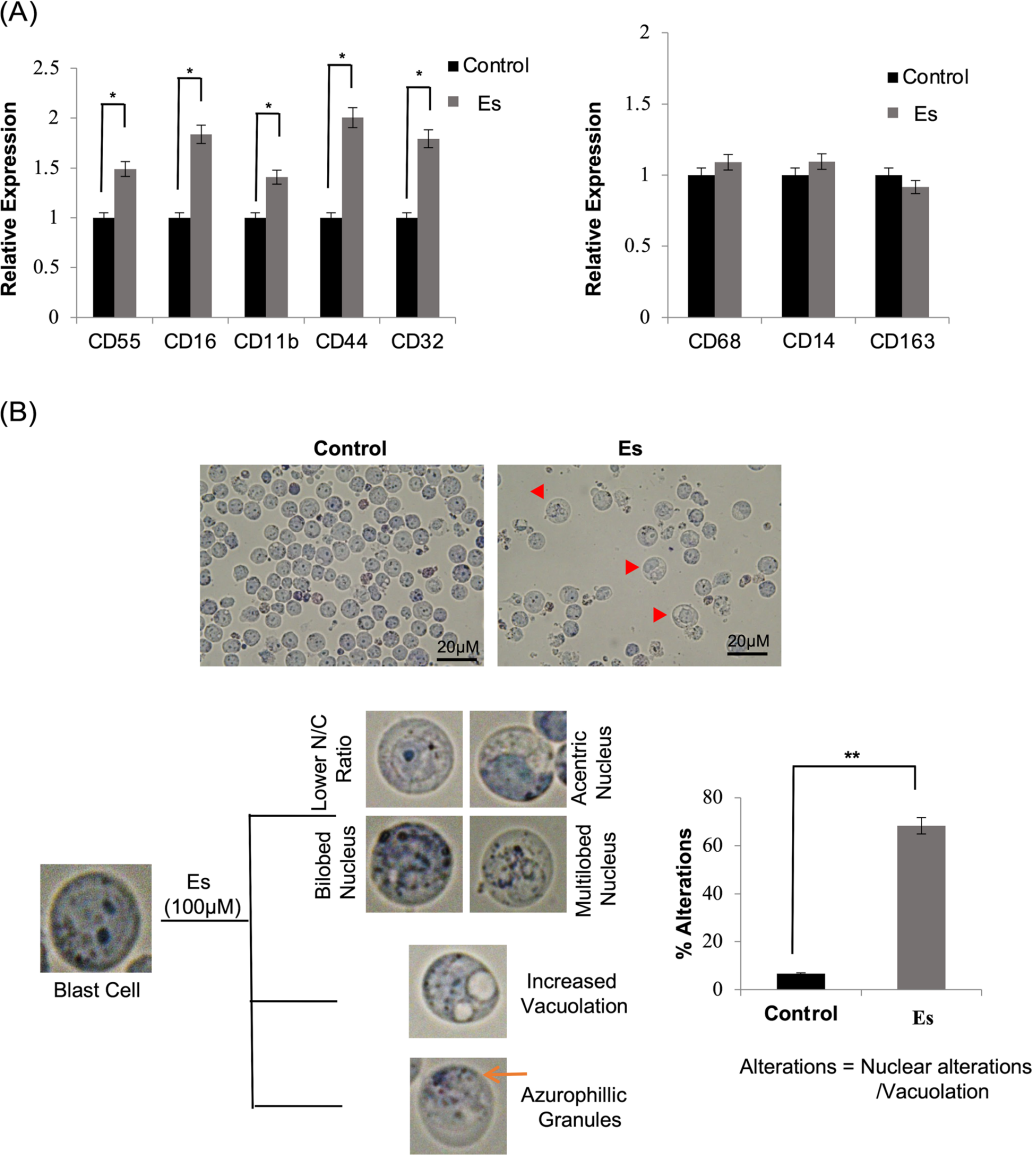
Terminal differentiation potential of esculetin. **A** Neturophilic (Left panel) and monocytic (right panel) specific surface marker analysis using flow cytometry to trace the leneage commitment following 96 h of 100 μM esculetin treatment. Data represents three independent experiments. (*p ≤ 0.05; **p ≤ 0.02). **B** Microscopic analysis of Wright-Giemsa-stained cells following 96 h of esculetin treatment (magnification: 60X). A total of 500 randomly selected cells were analyzed for nuclear alterations and appearance of vacuolation. Relative percentage alteration which includes bilobed, multilobed, acentric nucleus, cells with lower nucleocytoplasmic (N/C) ratio and/or presence of vacuolation were plotted (right panel). Data represents three independent experiments. (*p ≤ 0.05; **p ≤ 0.02)

The myeloblasts can be reprogrammed to trans-differentiate into either neutrophilic or monocytic lineage. Evaluation of the cell surface marker expression, recognising specific netrophilic/monocytic lineage committed cells revealed upto two-fold increased neutrophilic markers (CD55,Cd16,CD11b, CD44, CD32) in esculetin-treated cells (Fig. 3A). The unaltered monocytic lineage specific markers in esculetin-treated cells are suggestive of terminal differentiation of blast cells into neutrophils rather than monocytes upon esculetin treatment.

We used Wright-Giemsa staining to examine the morphological alterations. Most of the untreated cells exhibited a typical blast cell morphology with a high nucleo-cytoplasmic ratio (Fig. 3B). Conversion of large sized blast cells into relatively smaller cells with a granular cytoplasm corroborated the observations with the side scatter characteristic of esculetin -treated cells (Fig. 2B). During maturation, extremely plastic nature of the nucleus equips the phagocytic cell to perform biomechanical functions that accomplish fast migratory properties. Upon Giemsa staining, morphologically identifiable neutrophilic lineage committed cells were detected upon 96 h of esculetin treatment. Prominent nuclear alterations viz*.* presence of bilobed, trilobed and acentric nucleus along with increased cytoplasmic vacuolation in esculetin-treated cells ascertain induction of neutrophilic differentiation in Kasumi-1 cells upon esculetin treatment (Fig. 3B). These morphological alterations were analogous to the earlier reported alterations induced by ATRA in various studies.

### Esculetin mediated differentiation involves differential activation of canonical/non-canonical wnt signaling pathways

More recently, a shift from beta catenin-dependent canonical to independent Wnt signaling has emerged to provide proliferative advantages and induction of maturation arrest in leukemic cells in a cell context dependent manner.

**Fig. 4:**
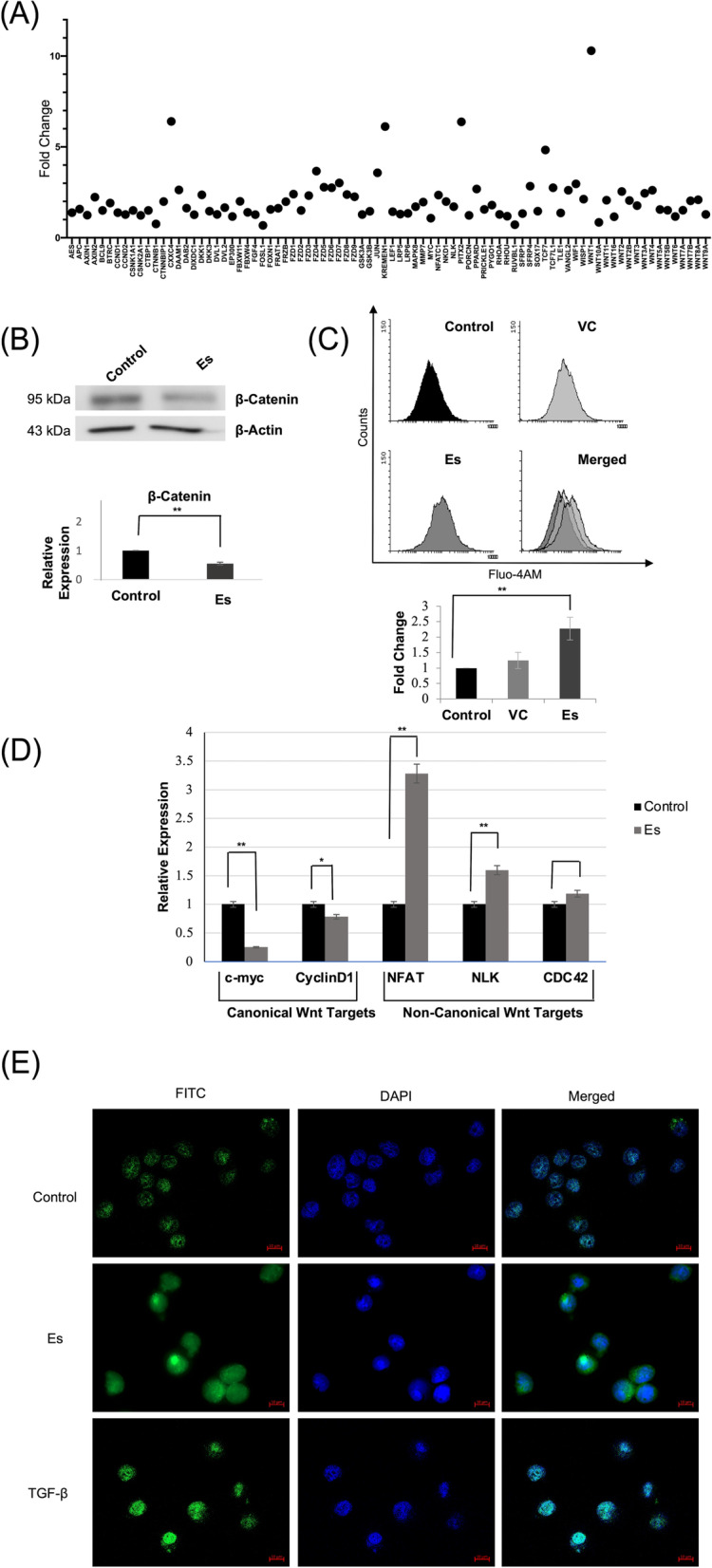
Differential Wnt axis activation upon esculetin treatment. **A** Relative gene expression profile of 84 canonical and non-canonical Wnt associated genes using RT2 profiler™ kit (Qigen corp). Cells were treated with esculetin for 24 h and were harvested to perform real-time PCR as per the manufacturer’s instructions. Untreated cells were taken as control. **B** Western blot analysis of β-catenin expression upon esculetin treatment. Total cell extract following 24 h of esculetin treatment was subjected to western blotting for β- catenin expression analysis. Representative blot of three independent experiments is shown. Densitometric analysis of relative β- catenin expression is shown in lower panel. (*p ≤ 0.05; **p ≤ 0.02). Full length immuno-blots are shown in supplementary data 1. **C** Intracellular calcium level analysis folowing 72 h esculetin treatment. Cells were harvested and stained with intracellular calcium binding dye -Fluo-4AM and analysed using flow cytometry. Relative fold change to untreated cells was plotted (lower panel). **D** Expression profiles of downstream targets of canonical and non-canonical axis using real time PCR following 48 h of 100 µM esculetin treatment. Data represents three independent experments (*p ≤ 0.05; **p ≤ 0.02). **E** Representative immunofluorescence images reveal a distinct distribution of β-catenin in esculetin treated/untreated cells (magnification 63X). Cells were fixed and stained with β-catenin antibody and FITC labelled secondary antibody. Representative images of three independent experiments are shown

We used RT^2^ profiler PCR array consisting of 84 Wnt associated genes to assess the canonical/non-canonical Wnt axes following 24 h of esculetin treatment. We found a significant downregulation of Wnt genes conferring stem cell self renweal (*FGF-4, SFRP-1, Wnt16*) while upto six-fold upregulation of differentiation associated genes (*DAAM-1, DKK1, KREMEN1, FZD5, PPARD and Wnt7B*) (Fig. 4A). Interestingly, the upregulated genes (*DAAM-1, DKK-1, KREMEN-1, PPARD and FZD5*) are majorly associated with the non-canonical axis of Wnt signaling pathway. We further verified the expression of Wnt genes found to be differentially expressed in the PCR array using qPCR to analyse the independent expression of the genes at the transcript level. The expression of these genes by qPCR was consistent with the findings of PCR array (Table 1) except *KREMEN1* and *FGF-4* that showed ~ sixfold higher upregulation and negligable expression respectively than the fold change observed in PCR array. In conjugation, downregulation of beta-catenin protein (Fig. 4B) was also observed. The stabilized beta-catenin enters the nucleus to activate canonical Wnt pathway. Incidentally, distinct fluorescent puncta of β-catenin were majorly detected within the nucleus with partial cytoplasmic staining in control cells. A relatively more diffused cytoplasmic β-catenin staining was observed in the esculetin treated cells. TGF-β was taken as a positive control which showed strong nuclear β-catenin staining (Fig. 4E). These observations suggest suppression of canonical Wnt pathway upon esculetin treatment.

**Table 1:** Gene expression profile of differentially expressed Wnt associated genes when analysed by RT2 profiler kit and varified by individual specific primers

	**S.No**	**Gene Symbol**	**Gene Name**	**Fold Change (RT2 Profiler Array)**	**Fold Change (Specific Primers)**
Differentiation	1.	*DAAM1*	Dishevelled associated activator of morphogenesis 1	2.63	2.47
	2.	*DKK1*	Dickkopf homolog 1 (Xenopus laevis)	1.54	2.01
	3.	*WNT7B*	Wingless-type MMTV integration site family, member 7B	1.94	2.18
	4.	*WNT2B*	Wingless-type MMTV integration site family, member 2B	2.06	2.44
	5.	*KREMEN1*	Kringle containing transmembrane protein 1	6.38	37.29
	6.	*PPARD*	Peroxisome proliferator-activated receptor delta	2.54	2.51
	7.	*FZD5*	Frizzled family receptor 5	3.13	3.16
	8.	*WISP1*	WNT1 inducible signaling pathway protein 1	3.20	3.87
Self-Renewal	9.	*FGF-4*	Fibroblast growth factor 4	0.42	0.000062
	10.	*SFRP1*	Secreted frizzled-related protein 1	0.54	0.73
	11.	*WNT16*	Wingless-type MMTV integration site family, member 16	0.54	0.22

The activated non-canonical Wnt/Ca^+2^ signaling attribute to enhanced intracellular calcium levels, we therefore, checked the intracellular calcium of esculetin-treated Kasumi-1 cells using Fluo-4/AM. Nearly two fold higher calcium levels were detected in cells following 72 h esculetin treatment (Fig. 4C).

To confirm the differentially activated canonical/non-canonical Wnt axis upon esculetin treatment, the expression of the target genes of both the axes were analysed at the transcript level. Downregulation of canonical Wnt targets (*c-MYC, CYCLIND1*) while upregulation of downstream non-canonical/Ca^+2^ associated genes (*NFAT, NLK, CDC42*) strongly suggest the activation of non-canonical Wnt signaling in Kasumi-1 cells upon esculetin treatment (Fig. 4D).

### Esculetin activates non-canonical/ Ca^+2^ Wnt axis through enhanced influx of ER Ca^+2^ pool

**Fig. 5:**
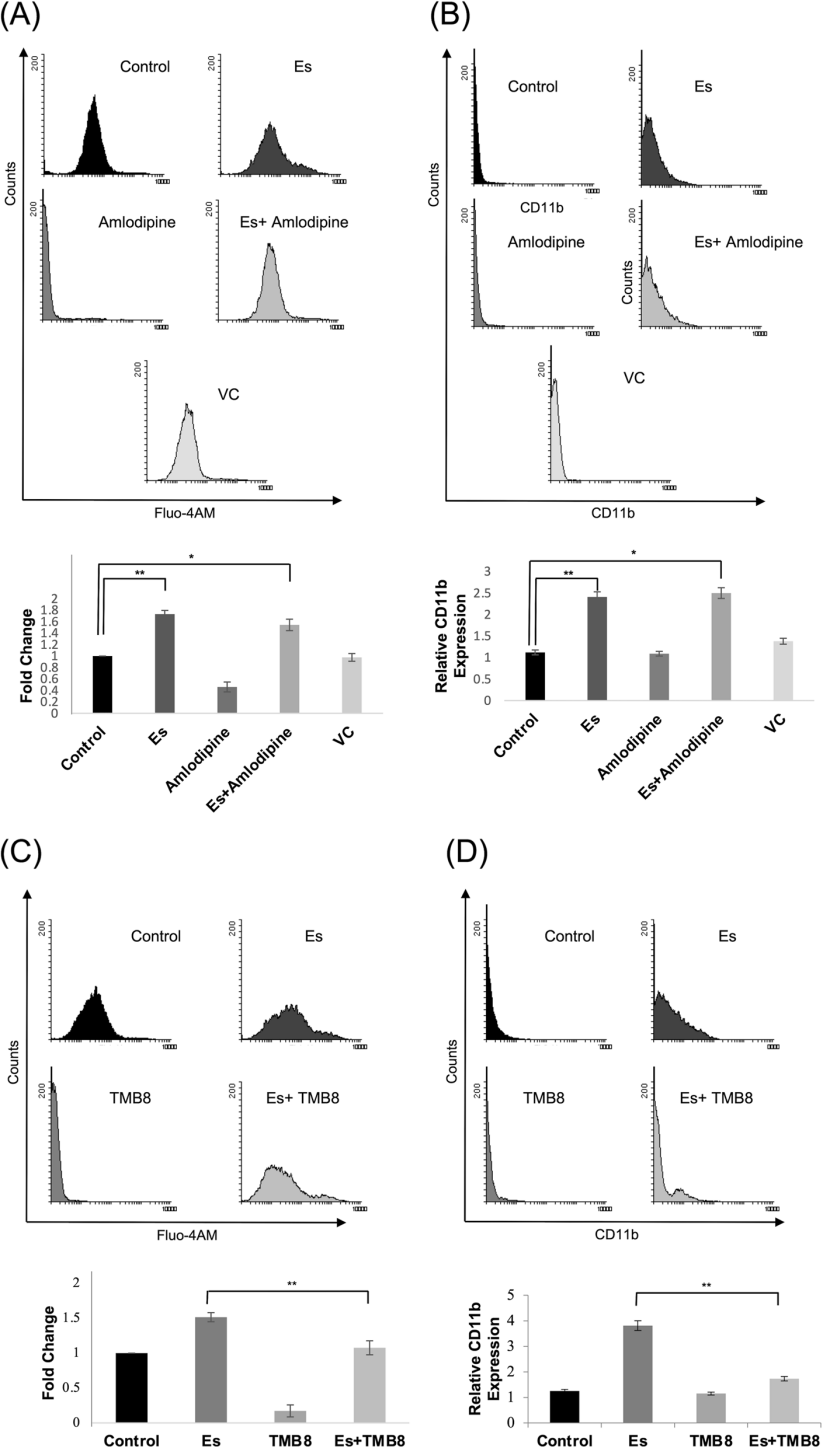
Differential uptake of calcium levels to induce blast cell differentiation. **A** Flow cytometric analysis of effect of amlodipine (membrane calcium channel blocker) on intracellular calcium levels. Cells were treated with esculetin alone or in combination with amlodipine for 72 h and stained with Fluo-4AM and subjected to flow cytometric analysis. A left shift in the fluorescence intensity demonstrate enhanced intracellular calcium levels in response to esculetin treatment. Relative fold change of three independent experiments were shown in lower panel. (*p ≤ 0.05; **p ≤ 0.02). **B** CD11b expression following 96 h of esculetin treatment alone or in combination with amlodipine. Relative CD11b expression of three independent experiments were shown in lower panel. (*p ≤ 0.05; **p ≤ 0.02). **C** Effect of TMB-8 (endoplasmic reticulum IP3R calcium channel blocker) on intracellular calcium levels following esculetin alone or in combination with TMB8 treatment. Cells were processed as mentined for amlodipine treatment. Relative fold change of three independent experiments were shown in lower panel. **D** CD11b expression alone or in combination with 96 h of esculetin treatment. Lower panel indicate relative CD11b expression of three independent experiments. (*p ≤ 0.05; **p ≤ 0.02)

Calcium is the central mediator of non-canonical/ Ca^+2^ axis. Upon Wnt activation, the cytosolic calcium is elevated either through the endoplasmic reticulum calcium pool or the extracellular calcium influx. We inhibited intracellular calcium levels using two calcium channel blockers. We used amlodipine which blocks the transmembrane calcium influx across the cell and observed that amlodipine alone was able to suppress the intracellular calcium levels in Kasumi-1 cells (Fig. 5A). However, in combination with esculetin, the intracellular calcium levels in amlodipine treated cells were found to be upregulated which were comparable to the cells treated with esculetin alone. The high calcium levels in these cells were also coupled with enhanced differentiation of blast cells as compared to amlodipine alone treated cells when detected with CD11b antibody (Fig. 5B).

Interestingly, inhibiting the ER-IP3R calcium channel with TMB8, the intracellular calcium levels drastically reduced when assessed by Fluo-4-AM (Fig. 5C). TMB8 was also found to suppress the esculetin mediated intracellular Ca^+2^ levels. The reduced intracellular Ca^+2^ levels were consistent with abrogated differentiation in esculetin-treated cells (Fig. 5D). These findings clearly demonstrate the involvement of ER Ca^+2^ pool rather than extracellular Ca^+2^ influx in esculetin mediated activation of non-canonical Wnt pathway akin to blast cell differentiation. DMSO (VC) alone was not found to alter the intracellular calcium levels as well as CD11b expression levels (Fig. 5A,B).

### TGF-β mediated suppression of non-canonical wnt axis abrogates esculetin mediated differentiation in Kasumi-1 cells

**Fig. 6:**
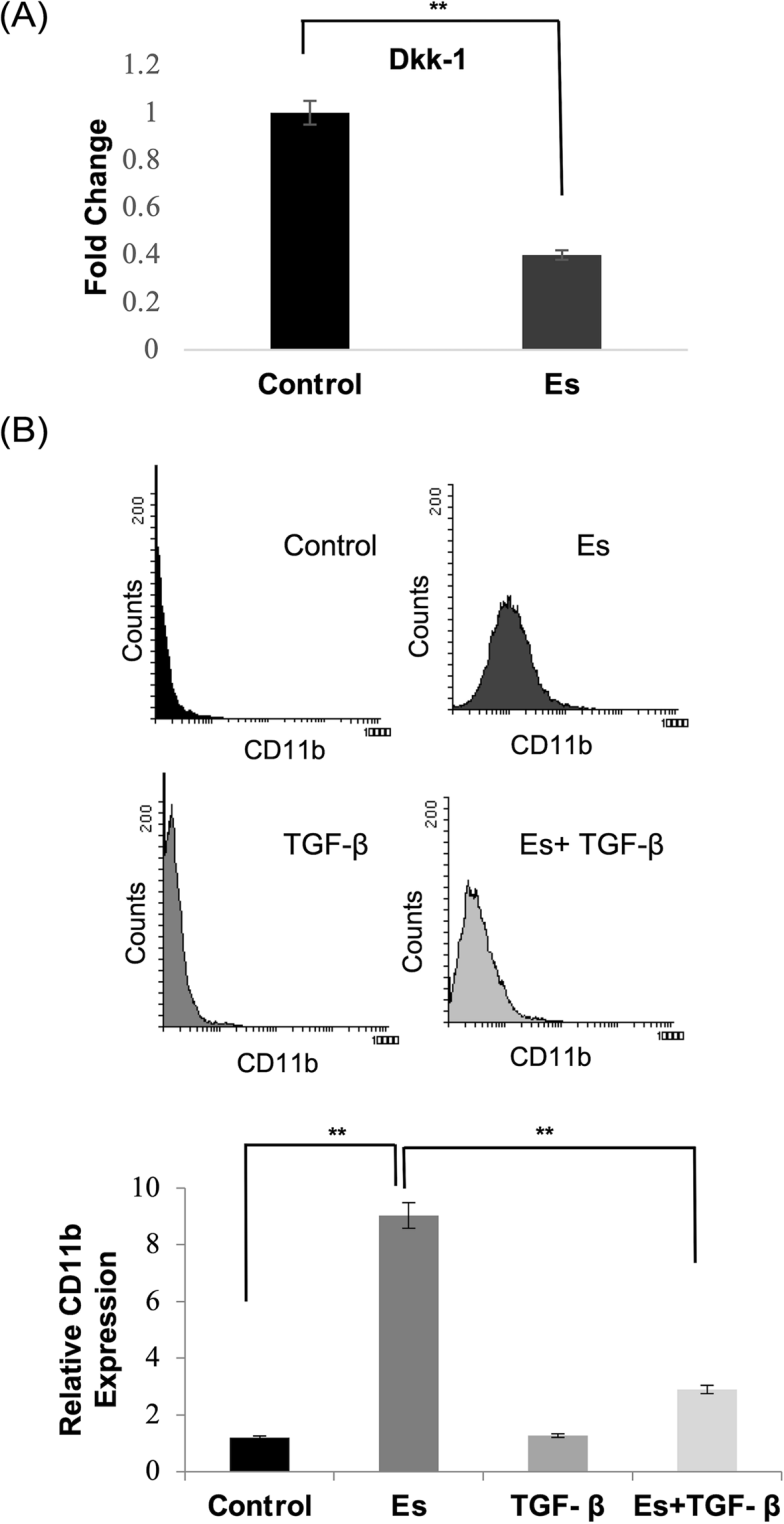
Alteration of esculetin mediated differentiation upon TGF-β exposure. **A** Real-time PCR analysis to check the expression of DKK-1 upon TGF- β (10 ng ml.^−1^) exposure for 24 h. (*p ≤ 0.05; **p ≤ 0.02). **B** Expression pattern of CD11b positive cell population following esculetin alone or in combination with TGF- β exposure. Lower panel represents relative CD11b expression of three independent experments and ± SE values. (**p ≤ 0.02)

To establish the definite role of non-canocal Wnt/Ca^+2^ axis in esculetin mediated differentiation, we further inhibited the axis using TGF- β which is known to abolish the non-canonical wnt signaling through downregulating the *DKK-1* expression (Fig. 6A). Of note, TGF- β was of particular interest for us because TGF- β exerts its effects by suppressing the non-canonical pathway while activating the canonical pathway. These properties of TGF- β antagonise the effects of esculetin that we observed during the current study i.e. activation of non-canonical/Ca^+2^ pathway and suppression of canonical pathway. Interestingly, consistent with the perivous studies, TGF- β was able to downregulate the *DKK-1*(~ threefold) expression in Kasumi-1 cells and suppress the esculetin mediate blast cell differentiation (Fig. 6B). A distinct nuclear localization of β-catenin puncta upon TGF- β exposure corroborated the involvement of TGF-beta in inducing canonical Wnt pathway (Fig. 4E).

### Suppression of canonical wnt axis by cardamonin augments esculetin mediated differentiation in Kasumi-1 cells

**Fig. 7:**
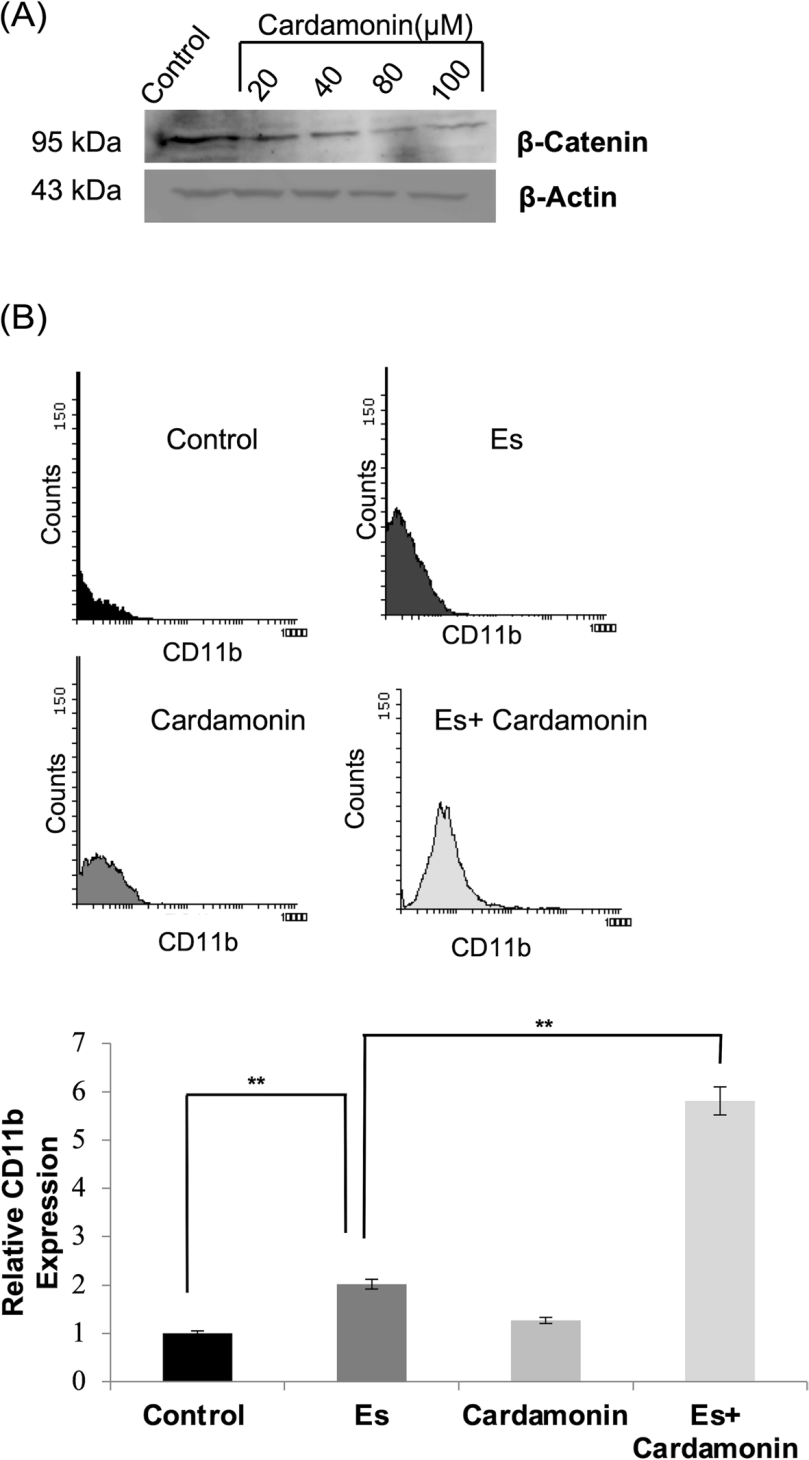
Effect of cardamonin on escultin mediated differentiation. **A** Westren blot analysis of expression pattern of β-catenin upon exposure to varied cardamonin concentrations (20,40,80,100 μM). Cells were harvested following 24 h of cardamonin exposure and total cell lysis was used to check the β-catenin expression levels using western bloting. Full length immuno-blots are shown in supplementary data 1. **B** Flow cytometric analysis of CD11b positive cells treated with cardamonin alone or in combination with esculetin. The experiments were performed in triplicates and ± SE values were plotted. Relative fold change to untreated cells was plotted (lower panel).The results show representative images (**p ≤ 0.02)

To determine the significance of the canonical Wnt axis in esculetin mediated Kasumi-1 blast cells, we abrogated the canonical Wnt axis by cardamonin, a commonly used Wnt signaling inhibitor which exert its effect by β-catenin degradation. We found downregulation of β-catenin protein with increasing concentrations of cardamonin in a dose dependent manner (Fig. 7A). At 80 µM cardamonin concentration, a drastically enhanced (~ fourfold) CD11b expression was observed in esculetin-treated cells as compared to cells treated with esculetin alone (Fig. 7B). These observations clearly indicate that not only activation of non-canonical Wnt/Ca^+2^ axis but supression of the canonical Wnt axis is required for esculetin-mediated differentiation of Kasumi-1 blast cells.

## Discussion

A new generation strategy to reprogram the leukemic blast cells to abolish its proliferative capacity and restore the terminal differentiation potential is highly desirable to improve the currently existing decades-old chemotherapeutics. Succeeding the breakthrough achievements of ATRA in APL, escalated efforts are being made to identify differentiating agents for a wider subset of leukemic patients. In the present study, we propose esculetin as a differentiating agent for AML using Kasumi-1cells. Furthermore, we show that the effect of esculetin might be regulated, at least in part, by the “axis shift” of the Wnt signaling pathway.

Among the chromosomal abnormalities in AML, t (8;21) accounts for 10–15% of the cases characterized as M2 phenotype in French-American-British (FAB) classification. *AML-ETO* has shown to promote leukemogenesis by activation of myeloid precursors and blocking differentiation in blast cells [13]. In the current study, the reversal of differentiation block by esculetin corroborates our previous findings which demonstrate that esculetin suppresses *AML-ETO/c-Kit* expression levels [5]. Recent evidence highlight the possibility that differentiation and apoptosis are independent events with different regulatory mechanisms, identification of new drug targets with improved predictable effects will be beneficial to modulate differentiation potential while enhancing AML blast sensitivity to drug-induced apoptosis. Functional evidence of induction of both differentiation, as well as apoptosis in the current study by esculetin, emphasise the fact that apoptosis and differentiation are interlinked events. Multiple studies have recently established molecular association between different phases of cell cycle and pluripotency of the cells [14]. The central observation driving this concept is that G1 phase cells majorly responds to specific cell survival as well as differentiation signals. Cell cycle regulation and pluripotency are considered to exist in a circular relationship in stem cells where disruption of one will affect the other, resulting in generally two outcomes; cell death and/or differentiation. In the present study, we found that 100 μM esculetin induces G1-S phase arrest coupled with suppression of cell proliferation (~ two fold) following 24 h treatment. Notably, no further proliferation inhibition was observed at later time points. Moreover, esculetin at 100 µM concentration was sufficient to induce early apoptosis (~ fourfold) 24 h post treatment which gradually increased up-to 48 h with nearly all doses in dose dependent manner. These observations points towards induction of G1 phase specific lineage priming mechanisms upon esculetin treatment. In accordance, we also observed esculetin mediated gradual increase in early apoptotic cells up to 48 h of treatment. However, only a fraction of this population proceeded into late stages of apoptosis and undergoes cell death (Supplementary Fig. 1). These data suggests the possibility that cells that escaped the early apoptosis program were stimulated to undergo differentiation. Although detailed investigation is required to fully elucidate this observation. The capacity of Kasumi-1 cells to acquire more differentiated phenotype upon esculetin treatment was demonstrated by enhanced granularity and reduced cell size coinciding with elevated ROS levels. Quite interestingly, at 100 µM (IC50) esculetin concentration, nearly 49% cell population exhibited smaller cell size coupled with reduced CD34 (~ 40%) and enhanced CD38 (~ 39%) expression levels which strongly suggest induction of differentiation in the remaining cells following esculetin treatment. During maturation, extremely plastic nature of the nucleus equips the phagocytic cell to perform biomechanical functions that accomplish fast migratory properties [15]. A typical blast cell morphology with a high nucleo-cytoplasmic ratio upon esculetin treatment, converted to show varied phases of nuclear maturation akin to neutrophilic differentiation. Alterations in differentiation-antigen phenotype upon esculetin treatment provide a rational for achieving similar cyto-differentiating effects manifested by ATRA differentiation responsive leukemic cells.

The Wnt signaling is a central cascade governing the development and stemness, has also been explicitly implicated in both solid as well as liquid cancer cell plasticity. The β- catenin have shown to be essentially required for self-renewal of Leukemic Stem Cell (LSC) compared to Hematopoietic Stem Cells (HSCs) [16]. Thus, β- catenin have been considered as prognostics biomarker for AML aggressiveness [17, 18]. Interestingly, esculetin mediated suppression of Wnt β- catenin axis was accompanied by downregulation of stemness while upregulation of differentiation associated genes in Kasumi-1 cells (Table 1) highlight the suppression of Wnt β- catenin mediated aggressiveness in blast cells. More recently, aberrant methylation of antagonists of canonical Wnt axis have been detected in AML cells lines as well as patient samples [19, 20]. Moreover, accumulated evidences are in support of emerging role of non-canonical Wnt pathway during the leukemic stem cell differentiation which have long been considered as tumour suppressor pathway in variety of cells [21]. The activation of non-canonical Wnt axis have been observed to antagonize the canonical axis through β-catenin degradation. However, the underlaying mechanism is still unclear. Here, we demonstrate that esculetin elevate calcium release from the ER stores and concurrent upregulation of non-canonical/Ca^+2^ associated genes (*DAAM-1,DKK-1,KREMEN1,PPARD and FZD5*) and the downstream effectors (*NFAT,NLK,CDC42*) while inhibit the canonical Wnt axis by degrading β-catenin. The downregulation of canonical Wnt targets (*c-MYC and CYCLIN D1*) might explain the esculetin mediated growth suppression in Kasumi-1 cells. Dysregulated c-MYC and CYCLIN D1 have shown to be associated with aberrant tumour growth in many human cancers [22].

More recently, studies have shown that the transmembrane proteins KREMEN1 and KREMEN2 have high-affinity with DKK-1 receptors that functionally cooperate with DKK-1 to activate the non-canonical Wnt/ Ca^+2^ signaling [23]. Additionally, a well-known secretory protein TGF-β was shown to stimulate canonical Wnt signalling in p38-dependent manner by decreasing the expression of the canonical Wnt antagonist DKK-1 [24]. In the present study, a marked reduction in *DKK-1*expression levels upon TGF-beta exposure lead to a concomitant suppression of esculetin mediated differentiation in Kasumi-1 cells. Upon suppression of the canonical Wnt signaling by a well-established Wnt inhibitor cardamonin, a nearly fivefold upregulated CD11b markers strongly suggest the importance of the switching of canonical Wnt axis to non-canonical/Ca^+2^ axis upon esculetin treatment in inducing terminal differentiation of leukemic blast cells into phagocytic neutrophils.

## Conclusion

The present study demonstrates the potential of esculetin to release the maturation arrest and inducing neutrophilic differentiation in leukemic blast cell population. The study also highlights the importance of “axis shift” of the canonical to non-canonical Wnt signaling upon esculetin treatment which might abrogates the inherent proliferation to induce differentiation in leukemic blast cells. The current findings might provide further significant therapeutic interventions to consider esculetin as a potent differentiating agent in the treatment acute myeloid leukemia.

## Supplementary Information

**Additional file 1: Supplementary Table 1.** Primer sequences used to check expression of individual gene.

**Additional file 2: Supplementary Figure 1.** Dynamic induction of early/late phase apoptosis in Kasumi-1 cells upon esculetin treatment : Representative dot plot analysis of esculetin mediated apoptosis based on Annexin V and propidium iodide (PI) staining following 24 and 48h esculetin treatment. Annexin V positive cells were considered to undergo early apoptosis, and Annexin V + PI positive cells as late apoptotic cells. Percentage cell population in each quadrant and relative apoptosis in bar graph (right panel) are combined from three independent experiments. (*p ≤ 0.05; **p ≤ 0.02).

**Additional file 3.**

## Data Availability

The datasets used and/or analysed during the current study available from the corresponding author on reasonable request.
