# Supplementary material for: Esculetin releases maturation arrest and induces terminal differentiation in leukemic blast cells by altering the Wnt signaling axes
Source: BMC Cancer. 2023 May 1;23:387. doi: 10.1186/s12885-023-10818-1 (PMC10150528; doi:10.1186/s12885-023-10818-1)
Supplement: Supplementary file 1 — Additional file 1: Supplementary Table 1. Primer sequences used to check expression of individual gene. [file 12885_2023_10818_MOESM1_ESM.pdf]

Supplementary Table 1 Primer sequences used to check expression of individual gene.

S.N0	Gene	Primer	Sequence
1.	***DAAM1***	Forward Primer	CTCACAGCACAGCTCCATGA
		Reverse Primer	CACCAGGAGGTGGCATGATT
2.	***KREMEN1***	Forward Primer	TGTCCCAGGATGGACAGTCT
		Reverse Primer	GAAGCAGGAACACGATGGGA
3.	***WNT2B***	Forward Primer	CCGGACTGATCTTGTCTACTTT
		Reverse Primer	CACAGCACCAGTGGAATTTG
4.	***FGF4***	Forward Primer	CAGCAAGGGCAAGCTCTAT
		Reverse Primer	CTTGGTCTTCCCATTCTTGC
5.	***FZD5***	Forward Primer	AACCAGTTCAACCACGACAC
		Reverse Primer	CATAGGAAGAAGCGCAGGTC
6.	***DKK1***	Forward Primer	TGTTTGTCTCCGGTCATCAG
		Reverse Primer	TCCATGAGAGCCTTTTCTCC
7.	***PPARD***	Forward Primer	CTATCCGTTTTGGTCGGATG
		Reverse Primer	CGATGTCGTGGATCACAAAG
8.	***WNT16***	Forward Primer	GCAGAGAATGCAACCGTACA
		Reverse Primer	TCATGCTTTCACACCTCCTG
9.	***SFRP1***	Forward Primer	ATCTCTGTGCCAGCGAGTTT
		Reverse Primer	AGCCCCATTCTTCAGGTACA
10.	***WNT7B***	Forward Primer	ATCGAGAAGTCGCCCAACTA
		Reverse Primer	GCACGTGTTGCACTTGACAT
11.	***c-MYC***	Forward Primer	GGGTAGTGGAAAACCAGCCT
		Reverse Primer	GCAGCAGCTCGAATTTCTTCC

12.	***CYCLIND1***	Forward Primer	CCTCGGTGTCCTACTTCAAATG
		Reverse Primer	GATGGAGTTGTCGGTGTAGATG
13.	***CDC42***	Forward Primer	GATACTGCAGGGCAAGAGGATT
		Reverse Primer	CCCAACAAGCAAGAAAGGAGTC
14.	***NLK***	Forward Primer	CTGGATATTGAGCCGGATAGAC
		Reverse Primer	ATGTGTGGAGGTTGGAGTATG
15.	***NFAT***	Forward Primer	CCTGTCCCCTACGTCCTACA
		Reverse Primer	AGCGGCTCATTCTCCAAGTAG
