# Supplementary material for: Esculetin releases maturation arrest and induces terminal differentiation in leukemic blast cells by altering the Wnt signaling axes
Source: BMC Cancer. 2023 May 1;23:387. doi: 10.1186/s12885-023-10818-1 (PMC10150528; doi:10.1186/s12885-023-10818-1)
Supplement: Supplementary file 2 — Additional file 2: Supplementary Figure 1. Dynamic induction of early/late phase apoptosis in Kasumi-1 cells upon esculetin treatment : Representative dot plot analysis of esculetin mediated apoptosis based on Annexin V and propidium iodide (PI) staining following 24 and 48h esculetin treatment. Annexin V positive cells were considered to undergo early apoptosis, and Annexin V + PI positive cells as late apoptotic cells. Percentage cell population in each quadrant and relative apoptosis in bar graph (right panel) are combined from three independent experiments. (*p ≤ 0.05; **p ≤ 0.02). [file 12885_2023_10818_MOESM2_ESM.pdf]

Supplementary Figure 1

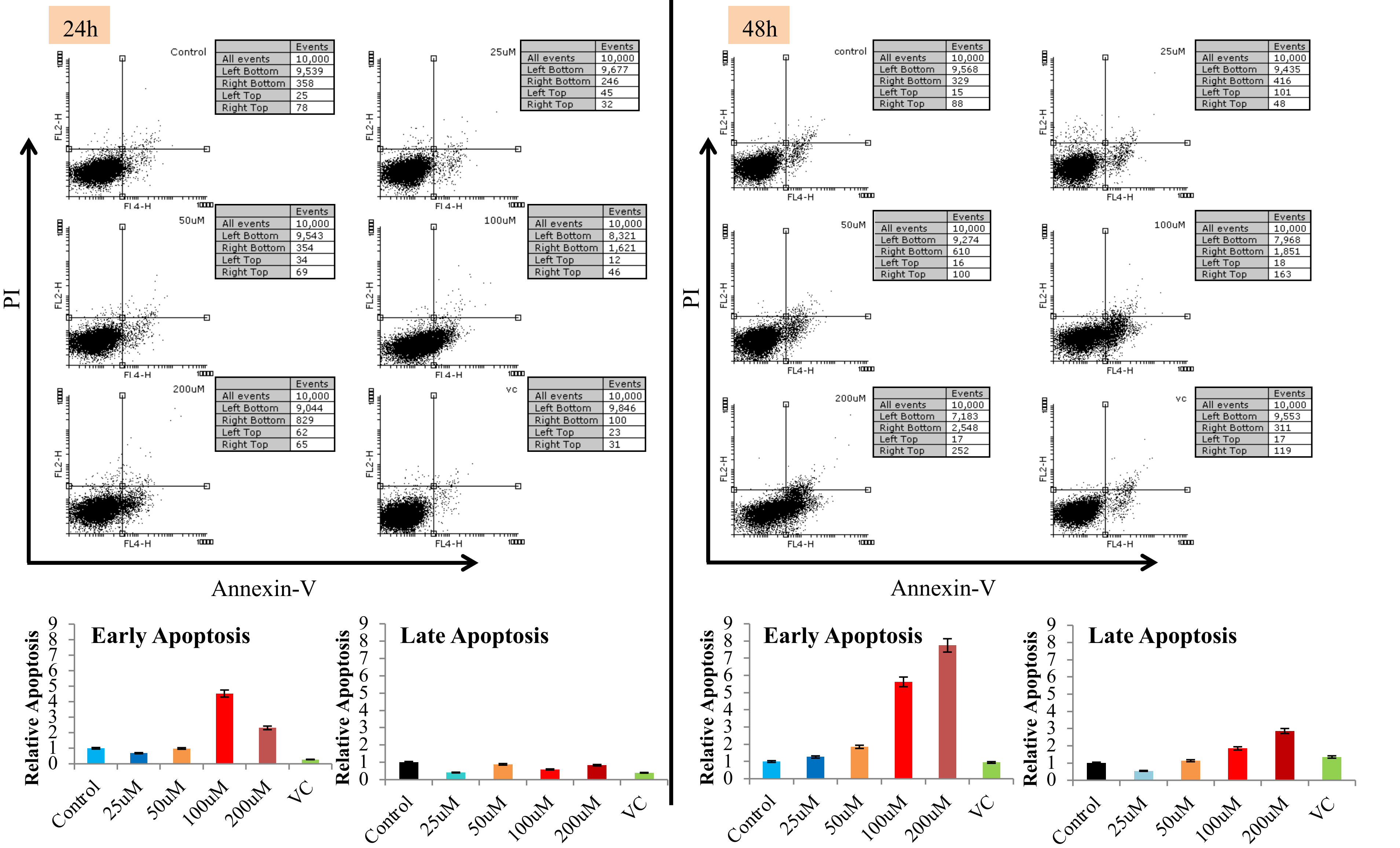

Supplementary Figure 1. Dynamic induction of early/late phase apoptosis in Kasumi-1 cells upon esculetin treatment : Representative dot plot analysis of esculetin mediated apoptosis based on Annexin V and propidium iodide (PI) staining following 24 and 48h esculetin treatment. Annexin V positive cells were considered to undergo early apoptosis, and Annexin V + PI positive cells as late apoptotic cells. Percentage cell population in each quadrant and relative apoptosis in bar graph (right panel) are combined from three independent experiments. (* $p \le 0.05$; ** $p \le 0.02$).
