# Supplementary material for: Esculetin releases maturation arrest and induces terminal differentiation in leukemic blast cells by altering the Wnt signaling axes
Source: BMC Cancer. 2023 May 1;23:387. doi: 10.1186/s12885-023-10818-1 (PMC10150528; doi:10.1186/s12885-023-10818-1)
Supplement: Supplementary file 3 — Additional file 3. [file 12885_2023_10818_MOESM3_ESM.pdf]

Figure 4 (B)

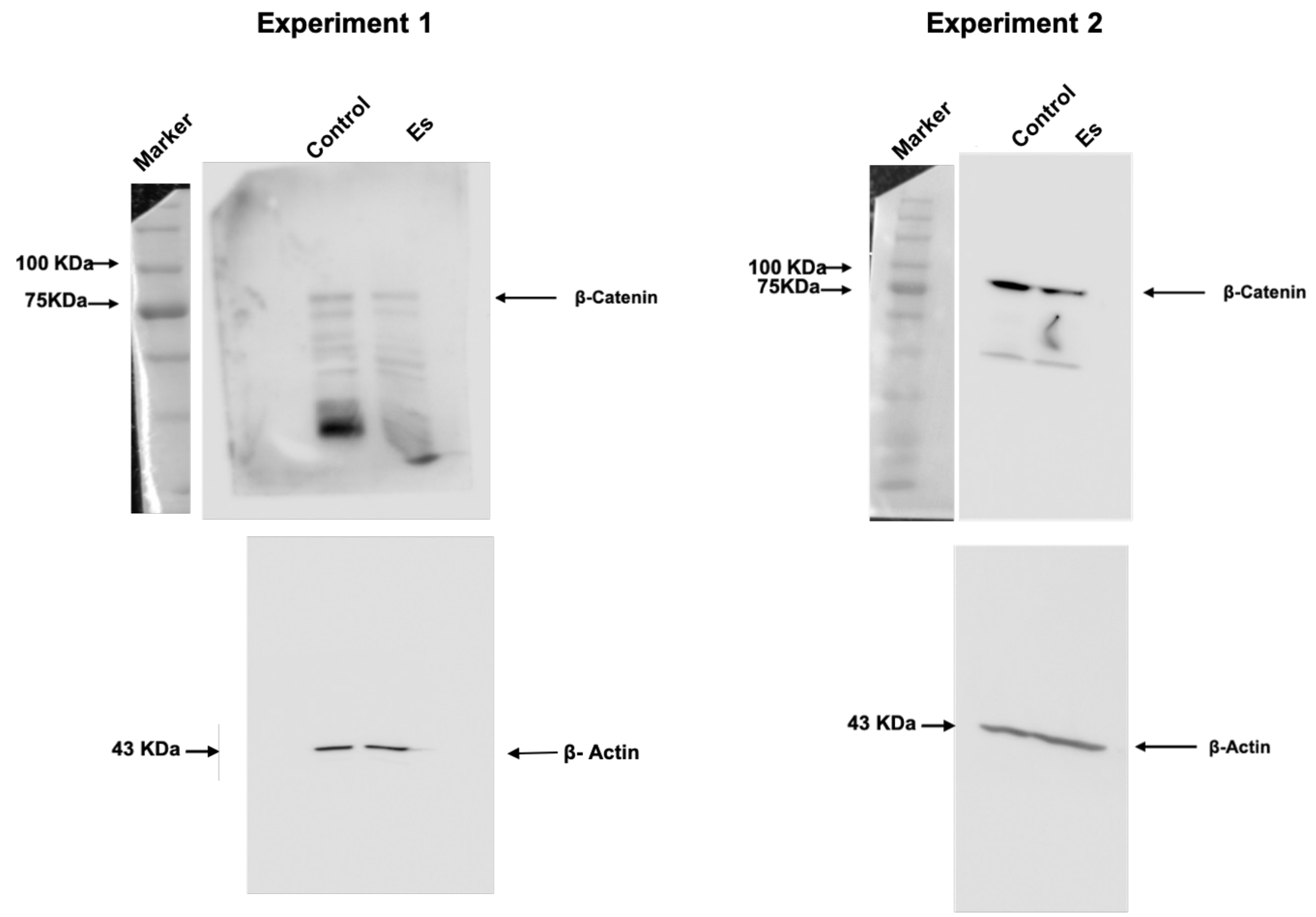


Immuno-blots of two independent experiments showing β-Catenin expression in esculetin treated/untreated cells.

Figure 7(A)

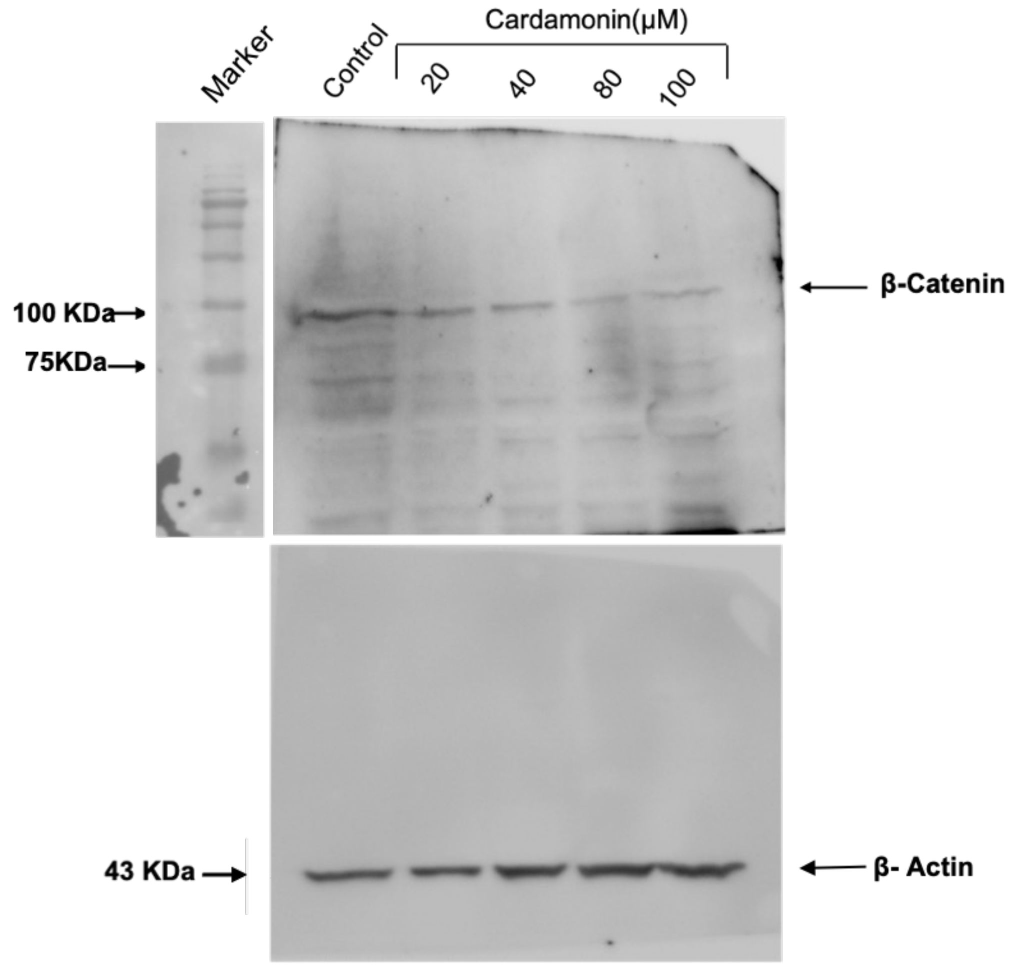

Marker
Control
Cardamonin(μM)
20
40
80
100
100 KDa
75KDa
β-Catenin
43 KDa
β- Actin
